# Non-Native Interactions Are Critical for Mechanical Strength in PKD Domains

**DOI:** 10.1016/j.str.2009.09.013

**Published:** 2009-12-09

**Authors:** Julia R. Forman, Zu Thur Yew, Seema Qamar, Richard N. Sandford, Emanuele Paci, Jane Clarke

**Affiliations:** 1 Cambridge University Chemical Laboratory, MRC Centre for Protein Engineering, Lensfield Road, Cambridge, CB2 1EW, UK; 2 Institute of Molecular and Cellular Biology, University of Leeds, Leeds, LS2 9JT, UK; 3 Cambridge University Department of Medical Genetics, Cambridge Institute for Medical Research, Addenbrooke's Hospital, Cambridge CB2 0XY, UK

**Keywords:** PROTEINS, CELLBIO

## Abstract

Experimental observation has led to the commonly held view that native state protein topology is the principle determinant of mechanical strength. However, the PKD domains of polycystin-1 challenge this assumption: they are stronger than predicted from their native structure. Molecular dynamics simulations suggest that force induces rearrangement to an intermediate structure, with nonnative hydrogen bonds, that resists unfolding. Here we test this hypothesis directly by introducing mutations designed to prevent formation of these nonnative interactions. We find that these mutations, which only moderately destabilize the native state, reduce the mechanical stability dramatically. The results demonstrate that nonnative interactions impart significant mechanical stability, necessary for the mechanosensor function of polycystin-1. Remarkably, such nonnative interactions result from force-induced conformational change: the PKD domain is strengthened by the application of force.

## Introduction

**Figure 1:**
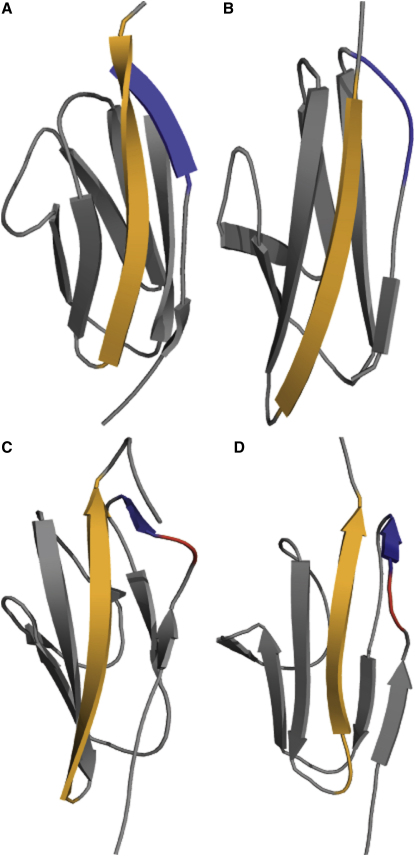
Structures of the Native and Force-Induced Intermediate States of PKD Domains (A) Native structure of the I27 domain from human cardiac titin (I27, Protein Data Bank [PDB] code 1tit [Improta et al., 1996]). (B) Native structure of human PKD domain (PKDd1, PDB code 1b4r [Bycroft et al., 1999]). (C) Native structure of archaeal PKD domain (ArPKD, PDB code 1l0q [Jing et al., 2002]). (D) Simulated mechanical unfolding intermediate of ArPKD. Although the native ArPKD has a short A′-strand (blue) interacting with the G-strand (orange), as seen in the I27 domain, PKDd1 apparently does not (as in fnIII domains). However, under applied force, both domains rearrange to form an intermediate. This intermediate (only ArPKD intermediate shown here) has nonnative interactions between residues in the loop close to the A′-strand (colored red) and the G-strand. These loop residues (13-15) were mutated to Pro to prevent formation of these putative nonnative H-bonds.

Diverse biological functions, including sensory pathways, development, and tissue elasticity, require proteins that resist unfolding under mechanical force. Experimental and simulation studies suggest that the mechanical strength of proteins is principally determined by the secondary structure and native topology of the force-bearing structural unit(s) (reviewed in Brockwell, 2007; Forman and Clarke, 2007; Oberhauser and Carrion-Vazquez, 2008): β-sheet proteins unfold at higher forces than α-helical proteins. Mechanical resistance is further modulated by the specific intramolecular interactions existing in the native state, such as the number of hydrogen bonds and the nature of the side-chain interactions (Best et al., 2003; Li et al., 2000; Craig et al., 2004; Ng et al., 2007; Borgia et al., 2008; Sharma et al., 2007). Immunoglobulin (Ig)-like domains, characterized by their β sandwich structure, with two antiparallel β sheets, are particularly resistant to unfolding under force. Furthermore, it appears that I-set Ig domains (such as the domain I27 from human titin) generally show greater force resistance than fibronectin type III (fnIII) domains (Oberhauser et al., 1998; Rief et al., 1998; Carrion-Vazquez et al., 2000; Li et al., 2005; Paci and Karplus, 1999). Although I-set and fnIII domains have a common Ig-like fold, they differ in the arrangement of the peripheral strands. In particular, the fnIII domains lack an A′-strand that forms hydrogen bonds to the G-strand in I-set domains such as I27 (Figure 1A). This A′/G-strand region of I-set domains confers mechanical resistance and has been called the “mechanical clamp” (Best et al., 2003; Li et al., 2000; Lu et al., 1998; Lu and Schulten, 1999).

The mechanical properties of polycystin-1 PKD domains (Forman et al., 2005; Qian et al., 2005) were first investigated due to their importance in polycystic kidney disease, a common Mendelian genetic disease (The International Polycystic Kidney Disease Consortium, 1994, 1995). Polycystin-1 was proposed to act as a mechanosensor, transducing fluid flow detected by the cilia of kidney epithelial cells into changes in intracellular calcium levels (Nauli et al., 2003). The extracellular portion of polycystin-1 is modular in nature, and β sandwich, Ig-like PKD domains comprise approximately 40% of the structure (Hughes et al., 1995; Sandford et al., 1997; Bycroft et al., 1999). Atomic force microscopy (AFM) experiments, applying force at the N and C termini of PKD domains, showed that these PKD domains resist unfolding under significant force, a requirement for their function as mechanosensors (Forman et al., 2005; Qian et al., 2005).

Surprisingly, these studies demonstrated that PKD domains are significantly more resistant to mechanical force than would have been predicted from their native structure. The first PKD domain from human polycystin-1 (PKDd1), the only polycystin-1 PKD domain with a known structure (Bycroft et al., 1999), shares the fnIII topology, lacking an A′-strand (Figure 1B). However, PKDd1 and PKD domains 2-4 (also from human polycystin-1) exhibit mechanical properties similar to those observed in I-set Ig domains, including I27 (Forman et al., 2005; Qian et al., 2005; Li et al., 2002). In this earlier study, molecular dynamics simulations were carried out to investigate the forced unfolding of PKDd1. The average unfolding forces in the steered molecular dynamics (SMD) simulations were similar for I27 and PKDd1, in agreement with the experimental results (Forman et al., 2005).

Importantly, the simulations suggested that PKDd1 undergoes a rearrangement in the A-B loop region under force. In the native state there are two hydrogen bonds between the A-B loop and residues at the end of the G-strand. The intermediate shows an increase in the number of interactions between the A-B loop and the G-strand; there are additional nonnative hydrogen bonds and side-chain/side-chain contacts (Figure 1D). This is reminiscent of the A′-G interactions that act as the mechanical clamp in I27. In the simulations, these A-B loop/G-strand interactions are broken when PKDd1 reaches the transition state for unfolding, and after this point unfolding proceeds without any significant barriers. This resembles the unfolding of I27 where breaking the A′-G contacts triggers unfolding (Best et al., 2003; Li et al., 2000; Lu et al., 1998).

To explicitly test the hypothesis suggested by these simulations, i.e., to determine if nonnative contacts prevent PKD domains from unfolding under moderate force, a mutational analysis is required. However, PKDd1 is only marginally stable thermodynamically (Δ*G*_U-N_ ∼1-2 kcal mol^−1^) and thus unlikely to tolerate mutations. For this reason, we chose to investigate the mechanical properties of an archaeal PKD domain (here called ArPKD, Figure 1C) (Jing et al., 2002), which is structurally similar to PKDd1 but considerably more stable. This enables us to study the mechanical properties of mutants, to determine the mechanism of force resistance in PKD domains (Ng et al., 2007, 2005; Craig et al., 2001).

Analysis of the ArPKD structure (Jing et al., 2002) shows that it has more contacts between the A-B loop and the G-strand (Figure 1C), suggesting that PKD domains may indeed have an A′-strand, forming a stretch of parallel β sheet with the G-strand, like the I-set Ig domains. Comparing the structures of PKDd1 and ArPKD, they are very similar in this region, although there are fewer hydrogen bonding interactions in PKDd1. The PKDd1 structure is a nuclear magnetic resonance (NMR) structure (Bycroft et al., 1999), whereas the ArPKD structure was solved by X-ray crystallography; perhaps the human PKD domain has an A′-strand that was undefined due to insufficient NMR restraints in this region.

This structural analysis of the ArPKD domain raised the possibility that in the PKD domains the native A′-strand is itself responsible for the high mechanical strength of the domain, and not the nonnative interactions proposed previously. Here we use simulations and a careful choice of mutations to demonstrate that nonnative interactions are key for the robust mechanical properties of PKD domains.

## Results

Note that the ArPKD structure used here was a domain isolated from an archaeal surface layer protein of *Methanosarcina mazei*, residues 302–384 (here numbered 1–83) of structure 1L0Q.pdb (Jing et al., 2002).

### Simulations Suggest Nonnative Interactions in ArPKD Domains under Force

**Figure 2:**
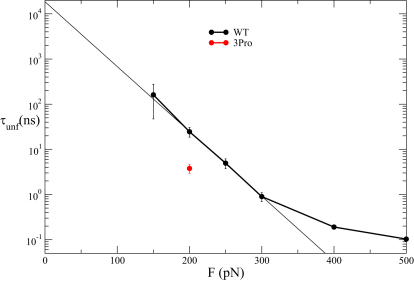
Force Modulation of Unfolding Time Average unfolding time at various forces for wild-type and 3Pro forms of ArPKD computed from forced unfolding simulations. At forces ≤ 300 pN there is an exponential decrease in the unfolding time with force (straight line is a fit of the data). The curvature at higher forces suggests that at very high forces the free-energy barrier for unfolding becomes negligible and the unfolding is dominated by the internal and solvent friction. The wild-type protein is mechanically stronger than the 3Pro mutant. Error bars correspond to ± 1 standard deviation.

Simulations were performed by applying a constant force of 100, 150, 200, 250, 300, 400, and 500 pN. At least 20 simulations were performed for each force, except at 100 pN and 150 pN where four much longer simulations (up to 300 ns) were performed. The average unfolding time as a function of the applied force is shown in Figure 2. As expected, the higher the applied force, the lower the average unfolding time. All unfolding events at forces ≤ 300 pN showed the same general features: formation of one or more force-induced intermediates with nonnative hydrogen bonds, followed by the breakage of these nonnative interactions and full unfolding. The robustness of the mechanism at forces below 300 pN is confirmed by the exponential dependence of the unfolding time on the force.

**Figure 3:**
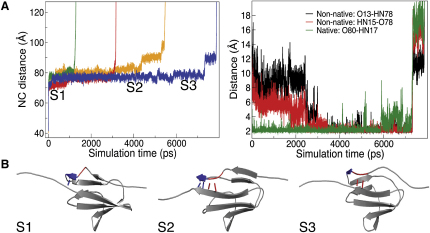
Unfolding Mechanism of ArPKD (A) Left: Plot of N-C extension (d_nc_, Å) against simulation time (ps) as ArPKD is pulled with a constant force of 200 pN in SMD simulations (data from four different simulations shown). The d_nc_ of the native state is ∼40 Å. The N to C extensions corresponding to the major states, S1-S3, along the unfolding pathway of ArPKD are indicated. In some of the simulations, an additional state with d_nc_ ∼90-100 Å appears just prior to full unfolding (see, for example, blue line). The lifetime of this state is much shorter than the unfolding time, which is dominated by states S2 and S3. As such, this minor state is unlikely to be detected experimentally and merely reflects the increased resolution of the simulations. Right: Hydrogen bond formation as a function of the simulation time for one of the simulations. The native HN17 to O80 hydrogen bond (green) lengthens and breaks before the unfolding event, but new nonnative hydrogen bonds form between residues 13 (black) and 15 (red) in the new A′ strand and residue 78 in the G-strand, which only break when the protein unfolds. (B) Structures of the major states, S1-S3, along the unfolding pathway of ArPKD in simulations where a constant force of 200 pN is applied to the termini of the protein. Native and nonnative hydrogen bonds, including the regions that are involved, are colored blue and red, respectively.

We analyzed in detail the mechanism of unfolding at the lowest force for which unfolding is complete in all 20 simulations (200 pN) since this is the regime closest to that explored by AFM experiments. A representative unfolding trajectory is shown in the left panel of Figure 3A; the unfolding process is complex and consists of four stages and three distinct intermediate states, labeled S1-3 in Figure 3B. First, the unstructured C-terminal region of the protein (residues ∼82–90) becomes fully extended accounting for the initial ∼40 Å to ∼75 Å step seen in Figure 3A. The core of the protein remains well structured and stable (S1, Figure 3B).

Second, the A/A′-strand residues are pulled toward the N terminus, causing them to slide along the G-strand. A subtle reorientation of the A/A′ strands and A-A′ loop also occurs, allowing residues 13 and 15 to form nonnative H-bonds to the G-strand (S2, Figure 3B).

Third, the native A′-G interactions are broken. Full unfolding, however, is prevented by the nonnative interactions made by residues 13 and 15 to strand G (S3, Figure 3B). In some cases, the presence of these interactions allows transient reformation of the native A′-G interactions, further increasing mechanical resistance. It should be noted that the second and third states, S2 and S3, are nearly degenerate in terms of d_nc_ (∼77 Å and ∼85 Å respectively, left panel of Figure 3A).

The strong nonnative interactions between the A-A′ region and the G-strand persist until they separate, leading to complete unfolding.

The formation and breakage of the native and nonnative hydrogen bonds described above is depicted by the time series of the distances between the various hydrogen bonding pairs shown in the right panel of Figure 3A.

**Figure 4:**
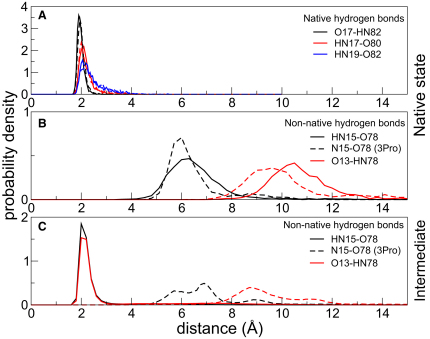
Native and Nonnative Hydrogen Bonds in Wild-Type and 3Pro Forms of ArPKD Probability distributions of distances between various main-chain hydrogen bonding pairs computed from the wild-type and 3Pro simulations. The distance considered is that between the amide hydrogen atom and the carbonyl oxygen atom (typical H-O distance for a hydrogen bond is 1.8–2.5 Å). For residues 15 in the 3Pro mutant, the distance between the amide nitrogen atom and the carbonyl oxygen is considered. An N-O distance of < 3.5 Å is considered to be favorable for hydrogen bond formation; we consider it here only as a measure of possible electrostatic interactions. Solid lines represent the wild-type protein and dashed lines represent the 3Pro mutant. (A) Native hydrogen bonding pairs from 2 ns equilibrium simulations. The dashed lines for 3Pro cannot be seen clearly as the 3Pro distributions overlap with the wild-type distributions. (B) Nonnative hydrogen bonding pairs from 2 ns equilibrium simulations. (C) Nonnative hydrogen bonding pairs for conformations with extensions 75 Å < d_nc_ < 90 Å extracted from all the pulling simulations at 200 pN.

To show that nonnative hydrogen bonds are only formed in the presence of force, we analyzed all 20 simulations at 200 pN, as well as the equilibrium simulation of the native states, and looked in detail at the propensity to form hydrogen bonds in the A-A′ region of the molecule in the presence and absence of force (Figure 4). To do so, the probability distribution of distances between hydrogen bonding pairs were computed from various simulations. The probability distributions for the native and nonnative hydrogen bonds were computed from an equilibrium 2 ns simulation of the native state (Figures 4A and 4B, solid lines) as well as for all structures with 75 Å < d_nc_ < 90 Å (i.e., structures with d_nc_ similar to S2-S3) extracted from the 20 simulations at 200 pN (Figure 4C, solid lines). A comparison of Figures 4A–4C (solid lines) indicates that the average behavior of all simulations conforms to the mechanism described in detail above.

### Choice of Mutations

The best way to test these observations is to make mutations in ArPKD that would destabilize the force-induced nonnative states observed in the simulations, and thus lower significantly the force required to unfold the protein. The simulations, which provide atomic-scale resolution, were used to guide our choice of mutations.

The principle observation from our simulations is that the native hydrogen bonds (between residues 17 and 19 and the G-strand) are broken and in the intermediate new nonnative hydrogen bonds are formed between residues 13 and 15 (in the A-A′ loop in the native structure) and the G-strand. On the basis of these data, a mutation was planned to introduce three prolines, replacing residues 13-15 (Thr, Ser, Gly). This mutant is called 3Pro from this point forward. These mutations, while they should allow native hydrogen bonds to remain, should prevent the nonnative hydrogen bonds from forming. Note that none of these residues form any hydrogen bonding interactions in the native state of ArPKD. As a control, a second mutation (2Pro) was also planned, to introduce prolines at positions 7 and 9, to measure any mechanical stability gained from the native state A-strand.

### Simulations of the 3Pro Protein

To check the predicted effect of insertion of the proline residues at positions 13-15, further molecular dynamics simulations were performed. Proline residues were substituted at these positions in the original structure of the wild-type protein and an equilibrium simulation of this mutated protein was performed for 2 ns. During this time the protein remained stably folded and importantly the native hydrogen bonds between the A and B strands and the A′ and G-strand were maintained (Figure 4A, dashed lines). Then 20 simulations of this protein were performed at a constant force of 200 pN. The unfolding pathway was very similar to that of wild-type i.e., a nonnative intermediate (d_nc_ ∼80 Å) was formed early in the simulations and unfolding took place from this nonnative intermediate (representative trajectories are shown in Figure S1 available online). However, this intermediate was distorted in the 13-15 region, compared with the intermediate seen in the wild-type simulations and, of course the nonnative hydrogen bonds that stabilize the intermediate in the wild-type were absent. The latter can be seen by comparing Figures 4B and 4C (dashed lines), which show that the probability distributions of the amide carbonyl distances in the equilibrium simulation and those at 200 pN are similar and centered far from hydrogen bonding distance.

The most significant result of these simulations is that the unfolding time was significantly lowered for the 3Pro mutant (3.8 ± 0.8 ns versus 24.6 ± 6.0 ns for wild-type, Figure 2), but because the native hydrogen bonds are largely intact in the 3Pro structure (Figure 4A, dashed lines) this cannot be ascribed to a weakening of the native state—it is most likely due to loss of the nonnative hydrogen bonds that stabilize the intermediate in the wild-type (Figure 4C, dashed lines).

### Mutant Monomer Thermodynamic Stabilities

Equilibrium denaturation experiments were performed on both ArPKD mutants to verify folding, measure thermodynamic stability, Δ*G*_U-N_, and quantify change in thermodynamic stability upon mutation, ΔΔ*G*_U-N_. Introduction of three proline residues in the A-A′ loop destabilized the protein by only 2.4 kcal mol^−1^ (wild-type Δ*G*_U-N_ = 4.3 kcal mol^−1^ versus 1.9 kcal mol^−1^ for 3Pro). However substitution by Pro of residues 7 (Asp) and 9 (Lys) in the A-strand was so destabilizing that the protein is no longer folded. This difference reflects the relative importance of these two regions in the stabilization of the native state.

### Atomic Force Microscopy Data

**Figure 5:**
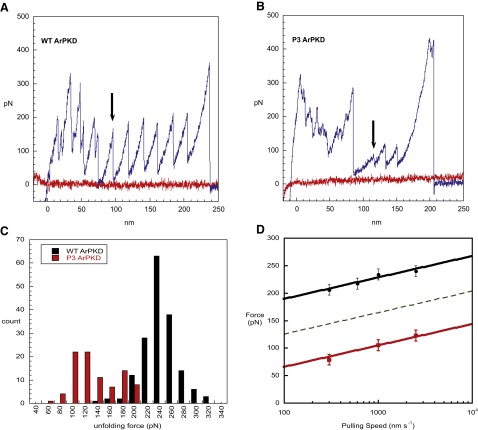
Unfolding Forces in Wild-Type and 3Pro Forms of ArPKD Sample AFM traces (A and B) show results from forced unfolding experiments on wild-type (WT) and 3Pro (P3) ArPKD, at 300 nm/s. The AFM traces of force versus distance (approach of tip, red line; extension of protein, blue line) show individual domains unfolding. The noise, seen at the start of the traces, is typical for AFM pulling experiments and is likely due to nonspecific tip-surface or protein-surface interactions. An unfolding event is characterized by the drop in force observed when the protein unfolds, extending suddenly in length and thus releasing the force on the cantilever. The first unfolding event in each trace is indicated by an arrow. Unfolding forces are measured from the height of the peak. The final peak represents the protein detaching from the tip. The wild-type trace (A) shows unfolding of six protein domains, whereas the 3Pro trace (B) shows unfolding of three domains, at significantly lower forces. (C) The unfolding forces (here at 1000 nm/s) are significantly lower for 3Pro (red) than WT (black). The dependence of the modal unfolding forces on the pulling speed (D) is the same for wild-type and 3Pro. However, the 3Pro mutant (filled red squares) unfolds at forces that are not only significantly lower than wild-type (filled black circles), but also significantly lower than would be predicted from the change in native-state stability (dashed line) (see Discussion, Supplemental Experimental Procedures, and Equation 1). This is consistent with our hypothesis that the 3Pro mutant disrupts nonnative contacts, formed under applied force, which are critical to the mechanical stability of ArPKD. Error bars correspond to ± 1 standard deviation.

AFM data were collected on polyproteins each containing 8 identical copies of wild-type or 3Pro domains. Collecting force spectroscopy data on 3Pro was very difficult. For wild-type approximately 1 in 30 AFM approach-retract cycles normally gives useful data, and on average each successful cycle shows four force peaks. For 3Pro, successful data collection occurred in a significantly lower proportion of approach-retract cycles, and showed fewer force peaks per trace. No changes in AFM protocol significantly increased data collection efficiency, so data collection was limited to three pulling speeds (300, 1000, and 2500 nm/s) to allow a reasonable amount of data to be collected. Compared with the wild-type data set, a larger proportion of traces showed unfolded protein domains. Upon completing data collection, small amounts of solid were visible in the protein sample, suggesting the protein had aggregated, likely hampering data collection. The AFM unfolding forces on wild-type and 3Pro unfolding were aggregated into histograms for each pulling speed (1000 nm/s) (Figure 3C), and the modes of the unfolding forces were calculated. Data were collected on 3 days for each construct. Data on 3Pro were collected over 2 additional days, to collect enough data at the 1000 and 2500 nm/s pulling speeds. The mean and mode unfolding forces at each pulling speed on each day are shown in Supplemental Experimental Procedures. 3Pro unfolds at significantly lower forces than wild-type at all pulling speeds (Figure 5). Note that variations in unfolding force between different days were similar to those observed on one day (Supplemental Experimental Procedures).

## Discussion

Simulations of the two different PKD domains suggest that they resist forced unfolding in the same way. Under force, simulations show that both the ArPKD and PKDd1 domains lose native hydrogen bonds in the A′-strand or the A-B loop, and undergo subtle rearrangements, resulting in formation of an intermediate state, which has similar structure to the native state, but with new nonnative hydrogen bonds to the G-strand that stabilize this intermediate (Figure 1D). The formation of force-stabilizing nonnative contacts in ArPKD is surprising because the native state of the ArPKD domain already shows interactions between the G and A′-strands. In the simulations of both PKD domains, the nonnative interactions prevent full unfolding even after the native A′-G interactions have broken, imparting remarkable mechanical resistance. Because it has significant thermodynamic stability, ArPKD was chosen to be the model system for experimental investigation of these nonnative interactions.

In a protein engineering analysis, one should generally avoid nonconservative mutations, such as mutations to proline (Fersht et al., 1992). Nonconservative mutations may cause structural distortions, and then it is not clear whether the local mutation or a global structural change is responsible for any observed effects of the mutation (Williams et al., 2003). However, if used carefully, proline mutations offer the only way to probe hydrogen bond deletion. The 3Pro mutant introduces prolines into a relatively unstructured region of the native structure, and not into a β sheet, where we would expect it to cause a major structural disruption (Randles et al., 2006). Although the 3Pro mutant is somewhat destabilized in comparison with the wild-type protein, it nevertheless allows the protein to fold. This is in contrast with the 2Pro mutant, which disrupts the hydrogen-bonding and packing interactions in the structured A-strand, and does not fold. This result is consistent with the fact that the 3Pro substitution is in a region that is less structured in the native state. This is confirmed by a 2 ns equilibrium simulation of the 3Pro mutant in native conditions. The native hydrogen bonds are unaffected by introducing the three proline residues into the unstructured loop.

Analysis of the wild-type and 3Pro simulations suggested that the unfolding pathway was the same for these proteins. This is consistent with what we see in the experiments. Importantly, the 3Pro and wild-type ArPKD unfolding forces exhibit the same dependence on the logarithm of the pulling speed, as illustrated in Figure 5D; the wild-type and 3Pro data are fit to lines of the same slope that fit the force data extremely well. For mutations that do not affect the dependence of unfolding force on pulling speed, the simplest explanation is that unfolding occurs via the same pathway, i.e., from the same starting structure and via the same transition state (Williams et al., 2003; Best et al., 2002). Thus, we can also conclude that the mutations destabilize, but do not completely abolish the nonnative intermediate.

It has been shown that the effect of a mutation on the unfolding force can be used to determine the change in free energy of the transition state (TS) relative to the ground state for unfolding (GS) (Best et al., 2002):

(1) $$\Delta \Delta G_{\text{TS-GS}}=Ax_{\text{u}}\left(F^{\text{wt}}-F^{\text{mut}}\right)$$

where *A* is Avogadro's number and *F*^wt^ and *F*^mut^ are the unfolding forces of wild-type and mutant proteins (at the same pulling speed) respectively, and where *x*_u_ is the distance of the transition state from the ground state, 0.27 nm (see Experimental Procedures).

In this case ΔΔ*G*_TS-GS_ for 3Pro is ∼5 kcal mol^−1^. This value is significantly greater than the observed destabilization of the native state (2.5 kcal mol^−1^). Thus the 3Pro mutation causes an extreme reduction in the mechanical stability of the ArPKD domain, far greater than might have been predicted from the effect of the mutation on native state stability (see detailed explanation in the Supplemental Experimental Procedures). We can, in fact, using Equation [1] predict the minimal unfolding forces which would be expected if the protein were unfolding from the native state (Figure 5D dashed line). The experimental unfolding force is significantly lower than would be predicted if the protein were unfolding from the native state. This is precisely what we would have predicted from the simulations: residues 13-15 are making stabilizing contacts (H-bonds with the G-strand) in the ground state for forced unfolding (the intermediate) that are not present in the native state. It is important to emphasize that this intermediate is not populated in the absence of force (see Supplemental Experimental Procedures); indeed no nonnative hydrogen bonds in the A-A′ region were observed in the MD simulations performed in the absence of force (Figures 4A and 4B, solid lines)—the formation of the nonnative intermediate structures is force induced (Figure 4C, solid lines). These nonnative H-bonds cannot form in the 3Pro mutant, destabilizing the intermediate significantly and thus drastically lowering the unfolding force. Nonnative interactions in the intermediate are apparently key for the mechanical stability of the ArPKD domain.

It is, perhaps, surprising that the ArPKD domain requires the formation of nonnative contacts for its mechanical stability. As mentioned earlier, we might have expected that the native A′-strand explained the mechanical stability in the PKD domains, as it does in the I-set domains of titin. However, it appears that these interactions are not responsible for establishing mechanical stability, and that the nonnative interactions centered around residue 14 are critical for mechanical strength in PKD domains.

### Conclusions

The data presented show that nonnative interactions form when the ArPKD domain is subjected to an external force applied at its N and C termini. These interactions are formed between the A-A′ loop and the G-strand, and appear to be responsible for maintaining structure in the domain under force, preventing unfolding. The simulation studies were essential to develop and refine a crucial hypothesis: that nonnative interactions are responsible for the mechanical properties of the PKD domain. Without these simulations, we would not have considered making mutations in residues 13-15 to probe the role of nonnative interactions. The same simulations showed that nonnative stabilizing interactions in the same region of the protein are responsible for the mechanical stability of the first human PKD domain from polycystin-1, which suggests that this may be a common mechanism for mechanical strength for this class of domains. It is interesting to note that simulations using native-centric models (such as Go models), which disregard enthalpy gain due to formation of nonnative contacts, cannot predict the existence of states stabilized by nonnative interactions.

Earlier work suggested that hydrogen bond and side-chain interactions in strands near the N and C termini were responsible for mechanical stability in Ig-like domains. The results presented here on PKD domains support these findings. However, our data also establish the importance of nonnative interactions. It was previously suggested that the I-set Ig domains were better able to resist unfolding under force than the fnIII domains due to the topological differences between these domains, namely the extent of interactions between the A′ and G-strands, near the N and C termini in the I-set domains. Yet although the first PKD domain from polycystin-1 shares the fnIII domain topology, PKDd1 unfolds at higher forces than any fnIII domain studied, and at forces higher than many Ig domains. The PKD domains seem to have a shared mechanism for resisting unfolding, based not on A′-strand interactions, but on nonnative interactions with the G-strand. These findings suggest that native state structure, and the topology it defines, may not be sufficient to predict the mechanical properties of proteins under force. Most importantly, these results highlight the importance of dynamical transitions and nonnative states in determining mechanical properties of proteins, which, as in the case of the PKD domain, are vital for its function. Because the formation of the nonnative interactions depends on the application of force, this means that the protein is paradoxically strengthened by the applied force, a phenomenon known as “catch-bonds.” Such behavior has been observed for the unbinding of protein-protein complexes (Evans et al., 2004; Thomas et al., 2002; Marshall et al., 2003; Guo and Guilford, 2006) but has not been observed previously for single domain protein unfolding. Our results could be an explicit elucidation of the manner by which catch behavior can be achieved.

It is interesting to note that PKD has a mechanical role in vivo like the systems where catch-bond behavior has been previously observed. One advantage of catch behavior is that it allows proteins to react quickly to changes in mechanical stress. In addition, it also allows proteins to exhibit a richer, “two-tier” response (e.g., catch state at lower forces and unfolded state at higher forces) depending on the magnitude of the force.

## Experimental Procedures

### Simulations

Simulations were performed using an all-atom model with implicit solvation (EEF1) (Lazaridis and Karplus, 1999), as previously described for the human PKD domain (Forman et al., 2005) (see also Supplemental Experimental Procedures). Simulations of the wild-type protein were initiated from the experimental structure (X-Ray structure 1L0Q for the archaeal domain) after a local optimization of the structure (100 steps steepest descent minimization). The 3Pro mutant was created from the crystal structure of 1L0Q by using the program CHARMM to build the proline ring from the pre-existing C-alpha coordinates of residues 13-15. The prolines were introduced one at a time and followed immediately by a local optimization (100 steps steepest descent minimization) of the resultant structure. A final energy minimization (500 steps steepest descent minimization) was then performed after all three proline substitutions were made. The root-mean-square deviation of the final structure with respect to the crystal structure 1L0Q was ∼0.5Å. Equilibrium simulations of 2 ns at 300K were performed to assess the stability of the wild-type and 3Pro structures with the force-field employed and to generate independent initial conformations for the simulations under mechanical force (see further discussion in Supplemental Experimental Procedures). Simulations were performed for each domain by applying a constant force of 100, 150, 200, 250, 300, 400, and 500 pN between the two termini, oriented as the vector joining them and in the direction of increasing distance, as in an AFM experiment. Twenty simulations were performed at each force except 100 and 150 pN where there were four simulations. Analogous simulations using steered molecular dynamics, where the force is applied through a spring with elastic constant 2000 pN nm^−1^ moved at constant speed (between 0.004 and 0.04 nm ps^−1^), were also performed and show the same unfolding mechanism described above; this highlights the robustness of the results.

### Cloning, Expression, and Purification

The ArPKD monomer gene was cloned into a modified pRSETA vector (Invitrogen). Standard site-directed mutagenesis reactions were used to introduce mutations into individual domains. The PKD multimeric constructs (ArPKD wild-type and 3Pro) were assembled according to previously established strategies (Steward et al., 2002; Ng et al., 2006). The proteins were expressed and purified as described previously (Ng et al., 2006). The two-step purification procedure involved Ni-affinity chromatography, followed by gel filtration. The N-terminal His-tag was not removed from the multimeric proteins.

### Thermodynamic Measurements

All experiments were carried out in phosphate-buffered saline (PBS [pH 7.4]) at 25°C. The stability of the individual ArPKD wild-type and mutant domains was determined by urea denaturation, using standard techniques (Pace, 1986). The protein was incubated for 3 hours in varying concentrations of denaturant and unfolding was monitored by change in intrinsic fluorescence. The change in free energy of unfolding for the mutant proteins, ΔΔ*G*_U-N_, was determined using mean *m*-values (<*m* >, 0.94 kcal mol^−1^ M^−1^) and the equation

[2] $$\Delta \Delta G_{\text{U}-\text{N}}=<m>\left({\left[\text{urea}\right]}_{50\%}^{\text{wt}}-{\left[\text{urea}\right]}_{50\%}^{\text{mut}}\right)$$

where [urea]_50%_ is the denaturant midpoint for wild-type (wt) and mutant (mut) proteins (Fersht, 1999).

### AFM Experiments

The AFM experiments were carried out in PBS (pH 7.4) at ambient temperature using an Asylum Research Molecular Force Probe as described previously (Best et al., 2001). For the ArPKD wild-type and mutant construct, data were collected at three different pulling speeds (300 nm s^−1^, 1000 nm s^−1^, and 2500 nm s^−1^) and data were also collected at 600 nm s^−1^ for wild-type. The resulting traces were analyzed as described previously (Rounsevell et al., 2004) and the unfolding forces recorded.

### AFM Data Fitting

The speed dependence of the unfolding force data was fit as described previously using PhiFit, software provided by Phil Williams (University of Nottingham), with the wild-type data, to calculate an *x*_u_ value (Ng et al., 2005). The fitting gives an *x*_u_ value of 0.27 nm. The mutant unfolding forces show the same speed dependence (i.e., the same *x*_u_) as the larger wild-type data set.

## Supplemental Data

Document S1. Supplemental Experimental Procedures, Five Figures, Two Tables, and Supplemental References
