# Supplementary material for: Non-Native Interactions Are Critical for Mechanical Strength in PKD Domains
Source: Structure. 2009 Dec 9;17(12):1582–90. doi: 10.1016/j.str.2009.09.013 (PMC2862302; doi:10.1016/j.str.2009.09.013)
Supplement: Document S1. Supplemental Experimental Procedures, Five Figures, Two Tables, and Supplemental References [file mmc1.pdf]

Supplemental Data

Non-Native Interactions Are Critical

for Mechanical Strength in PKD Domains

Julia R. Forman, Zu Thur Yew, Seema Qamar, Richard N. Sandford, Emanuele Paci, and Jane Clarke

(1) Simulations

(i) The use of implicit solvent in the simulations

The merits and disadvantages of using explicit or implicit solvents in mechanical unfolding simulations have been discussed previously [1]. While explicit solvent simulations can give insight into the role of water molecules in the unfolding process, the use of excessively large forces in these simulations may introduce artefacts related to the relaxation of water molecules [1], which in turn, may lead to an over-estimation of the importance of water [2].

The computational efficiency of implicit solvent models enables a larger number of simulations to be performed at much smaller forces. This allows statistically meaningful estimates of relevant quantities such as the average unfolding time to be made at conditions closer to AFM experiments. Here, the implicit solvent simulations of ArPKD were used to produce well defined hypotheses that were subsequently confirmed experimentally. The experimental verification of the simulations thus provides the best justification of solvent model used in the simulations.

(ii) Stability of the 2 ns equilibrium simulations

As can be seen from Figure S1, both the wildtype and 3Pro simulations enter a stable phase after the initial heating and relaxation phase (the first 0.5 ns). Importantly, the 3Pro simulations were as stable as that of the wildtype protein throughout the subsequent 2 ns of the simulation. Due to the use of a low friction coefficient (1 ps^{-1}) and an implicit solvent model, the extent of conformational sampling in the 2 ns simulations is higher than explicit solvent simulations of similar length.

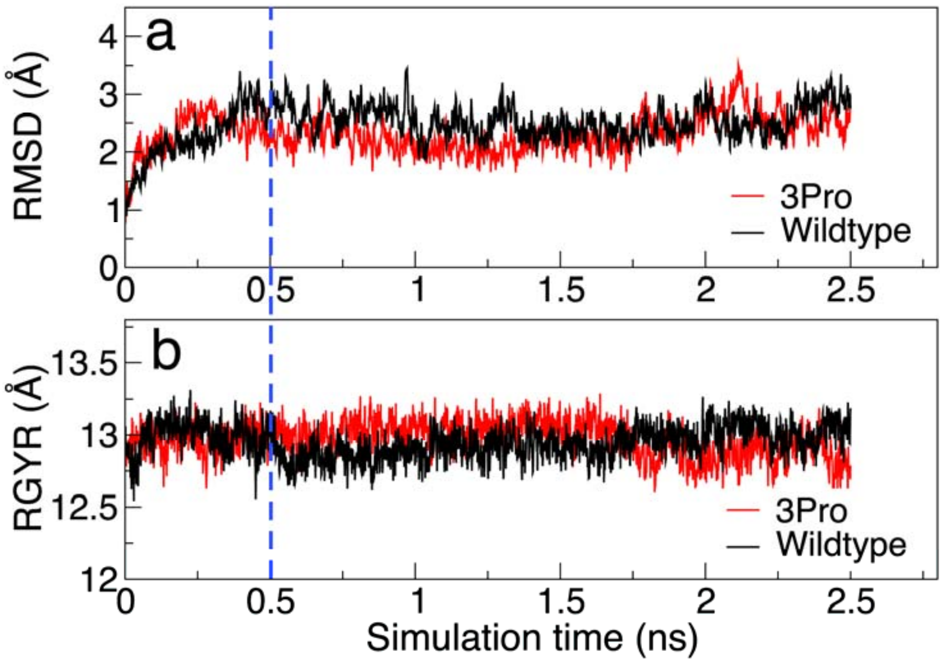

Figure S1. Equilibrium simulations

(A) C-alpha RMSD to the respective minimised starting structures and (B) C-alpha Radius of Gyration (RGYR) as a function of simulation time. The flexible C-terminal region (residues 82-90) was excluded from the RMSD and RGYR calculations to better reflect the stability of the structured core of the proteins during the simulations. The first 0.5 ns (dashed blue line) correspond to the initial heating and relaxation phase and were not used in the analysis or subsequent mechanical unfolding simulations.

(iii) Representative trajectories for the unfolding of the 3Pro mutant at 200 pN

The time series of the N-C extension of 3Pro (Figure S2) indicates that the mechanism of unfolding is similar to the wild-type protein where unfolding mainly occurs from states with d_{nc} ~80Å.

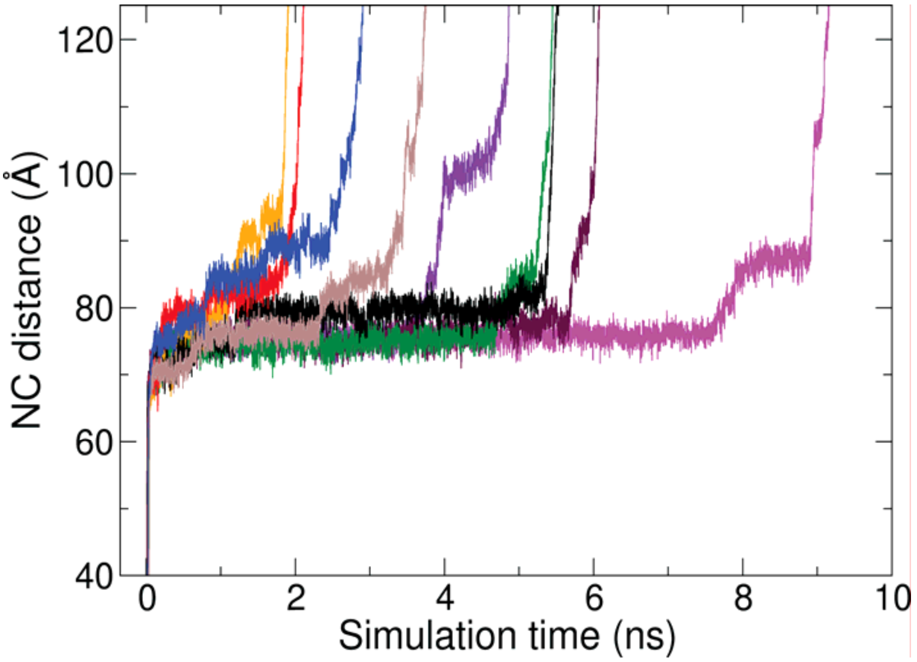

Figure S2.

Plot of the N-C extension (d_{nc}, Å) against simulation time (ns) as the 3Pro mutant was pulled with a constant force of 200 pN in SMD simulations (nine simulations shown).

(2) Protein engineering as a probe of forced unfolding landscapes

This analysis is based on protein folding Φ-value analysis [3] modified to describe forced unfolding (explained in detail in [4]).

In the simplest case (Figure 1) the protein will unfold from the native state (N) via the transition state (TS) to the forced unfolded state (U). The force required to unfold the protein is related not to the thermodynamic stability ($\Delta G_{U\text{-}N}$) but to the height of the energy barrier ($\Delta G_{TS\text{-}N}$). A mutation in a protein can lower the thermodynamic stability ($\Delta\Delta G_{U\text{-}N}$). If the mutation is in a region of the protein which is fully folded at the transition state, then the transition state will be destabilized to the same extent as the native state. The barrier to forced unfolding is unaffected ($\Delta G_{TS\text{-}N} = 0$) and the unfolding force of wild-type and mutant proteins will be the same (Figure S3a).

If a mutation is made in the protein at a site that is completely unfolded in the transition state then the stability of the TS will be unaffected, so the barrier to unfolding will be lowered by the same amount ($\Delta\Delta G_{TS\text{-}N} = \Delta\Delta G_{U\text{-}N}$, Figure S3b). Thus the force required to unfold the protein will be lower, by an amount that can be predicted [4]:

$$\Delta\Delta G_{TS-N} = A x_u \left(F^{wt} - F^{mut}\right), \qquad [1]$$

where A is Avagadro's number, x_u is the distance between N and the TS and F^{wt} and F^{mut} are the unfolding forces of wild-type and mutant proteins, respectively.

Thus in this simple 2-state case the $\Delta\Delta G_{U\text{-}N}$ (in the case of the 3Pro mutant this is 2.5 kcal mol^{-1}) predicts the *lowest* unfolding force of any mutant protein.

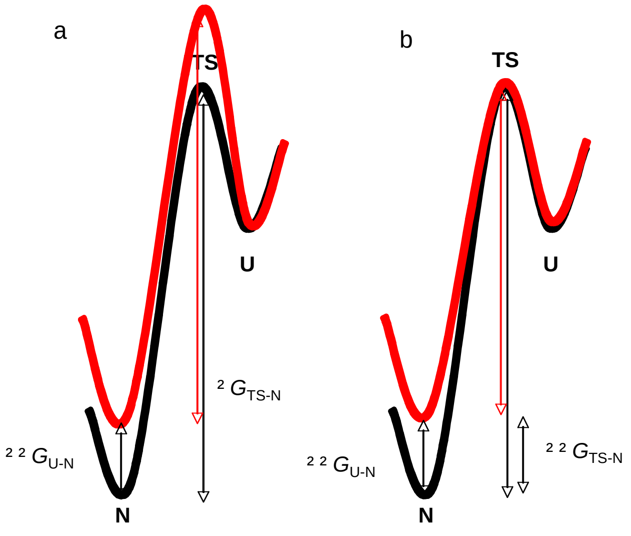

Figure S3. Changes in the energy landscape of a simple 2-state system on mutation
The unfolding force depends on the height of the energy barrier between the native (N) and transition states (TS) for unfolding ($\Delta G_{TS\text{-}N}$). (a) If the mutation is in a region of the protein which is fully folded in TS then $\Delta\Delta G_{TS\text{-}N} = 0$. (b) If the mutation is in a region of the protein which is fully unfolded in TS then $\Delta\Delta G_{TS\text{-}N} = \Delta\Delta G_{U\text{-}N}$. Thus change in stability on mutation ($\Delta\Delta G_{U\text{-}N}$) predicts the lowest unfolding force that can be expected on mutation.

In the 3Pro mutant of ArPKD the unfolding force is significantly lower than that predicted by the $\Delta\Delta G_{U\text{-}N}$ (see main text Figure 5d). The free energy barrier between the ground state (GS) for forced unfolding and the TS is destabilized by more than would be predicted from $\Delta\Delta G_{U\text{-}N}$. Thus the simple 2-state mechanism does not hold.

The simplest explanation is that the ground state for forced unfolding is destabilized more than the native state by the 3Pro mutation. i.e. the native state is NOT the ground state for forced unfolding. Our simulations show that under force an intermediate is populated, and this intermediate is the ground state from which the protein unfolds (Figure S4).

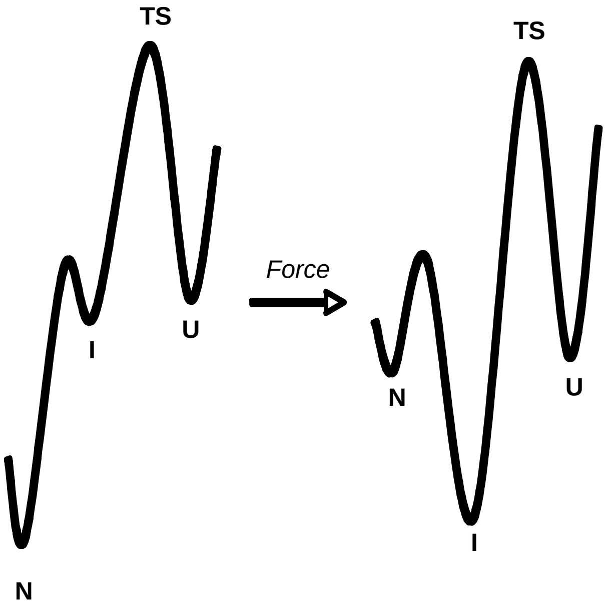

Figure S4. Hypothetical energy landscape of PKD domains under applied force
I is not populated in the absence of force. When a force is applied I is stabilised, relative to N and so becomes the most stable state. The ground state for forced unfolding is thus the intermediate I.

The simulations suggest that the region where the mutations have been made (residues 13-15) unfold at the transition state. Thus we concluded that intermediate is destabilized by approximately 5 kcal mol^{-1} (from use of equation [1]) by the 3Pro mutation. This is shown in Figure S5.

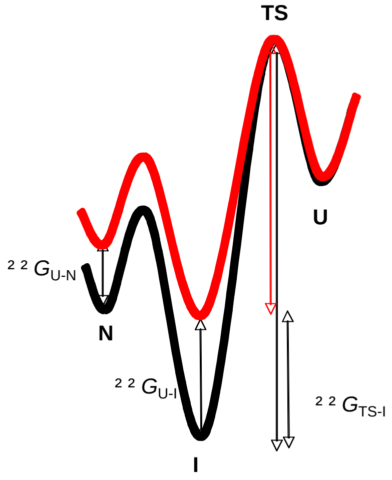

Figure S5. The effect of proline mutations on the hypothetical energy landscape of PKD domains under applied force

The ground state for forced unfolding is the intermediate I. This has non-native interactions so that I is destabilized more than N by the 3Pro mutation. Thus the change in unfolding force is greater than would be predicted from $\Delta\Delta G_{U\text{-}N}$.

Table S1. Forced unfolding simulations of wild-type and 3Pro ArPKD

Constant force applied (pN)	Number of simulations	Number of unfolding events	Mean unfolding time (ns)*	Standard Deviation*
Wild-type				
150	4	2	161	114
200	20	20	24.55	5.95
250	20	20	19.35	4.69
300	20	20	0.9	0.2
3Pro				
200	20	20	3.78	0.82

* The mean unfolding time and the standard deviation were computed using a maximum likelihood approach assuming single exponential unfolding kinetics [5]. This approach utilises information from all the simulations, including those that have remained folded.

Table S2. Average modal unfolding forces of ArPKD wild-type and mutants

Pulling Speed (nm/s)	300	1000	2500
ArPKD WT (pN)	206±8	234±6	240±14
3Pro (pN)	79±9	105±8	124±11

The errors quoted are standard deviations of the means of the results collected on different days.

Table S3. AFM unfolding forces for wild-type ArPKD

Pulling Speed (nm/s)	Mean (pN)	Mode (pN)	Standard Deviation	Number of Measurements
300	233	216	40	37
	205	203	29	63
	200	200	22	64
600	217	217	25	35
	240	222	57	56
	211	213	31	56
1000	240	240	27	64
	232	229	25	64
	226	232	32	41
2500	253	256	35	58
	243	231	38	85
	253	233	49	49

Table S4. AFM unfolding forces for ArPKD mutant 3Pro

Pulling Speed (nm/s)	Mean (pN)	Mode (pN)	Standard Deviation	Number of Measurements
300	82	83	19	18
	73	68	28	23
	80	85	20	17
1000	132	108	40	31
	115	112	31	25
	131 [a]	96	38	34
2500	154 [a]	134	48	45
	142	112	51	53
	155 [a]	127	53	35

[a] Data in this row are a combination of data collected on two days, as it was not possible to collect a sufficient amount of data on one day (see main text).

SUPPLEMENTAL REFERNCES

1. Paci, E., and Karplus, M. (1999). Forced unfolding of fibronectin type 3 modules: An analysis by biased molecular dynamics simulations. J. Mol. Biol. *288*, 441-459.
2. Ng, S. P., and Clarke, J. (2007). Experiments suggest that simulations may overestimate electrostatic contributions to the mechanical stability of a fibronectin type III domain. J. Mol. Biol. *371*, 851-854.
3. Fersht, A.R., Matouschek, A., and Serrano, L. (1992). The folding of an enzyme. I. Theory of protein engineering analysis of stability and pathway of protein folding. J. Mol. Biol. *224*, 771-782.
4. Best, R.B., Fowler, S.B., Toca-Herrera, J.L., and Clarke, J. (2002). A simple method for probing the mechanical unfolding pathway of proteins in detail. Proc. Natl Acad. Sci. USA *99*, 12143-12148.
5. Zagrovic, B., and Pande, V. (2003). Solvent viscosity dependence of the folding rate of a small protein: Distributed computing study. J. Comput. Chem. *24*, 1432-1436.
